# Childhood and adolescent cancer: early diagnosis challenges

**DOI:** 10.1590/1806-9282.2024S128

**Published:** 2024-06-07

**Authors:** Denise Bousfield da Silva, Mara Albonei Dudeque Pianovski, Maria Tereza Fonseca da Costa

**Affiliations:** 1 Universidade Federal de Santa Catarina, Brazilian Society of Pediatrics – Florianópolis (SC), Brazil.; 2 Universidade Federal do Paraná, Brazilian Society of Pediatrics – Curitiba (PR), Brazil.; 3 University Estácio de Sá, Brazilian Society of Pediatrics – Rio de Janeiro (RJ), Brazil.

## INTRODUCTION

Childhood and adolescent cancers differ from adult cancers in terms of both the type of normal cellular counterpart involved and the mechanisms underlying malignant transformation^1,2^.

Malignant neoplasms in children and teenagers predominantly affect blood cells and supporting tissues. Leukemia, central nervous system (CNS) tumors, and lymphomas are most observed in this age group. However, the global incidence of these conditions varies significantly, largely influenced by demographic and socio-economic factors in the regions under study^1,2^.

Contrary to adult cancers, childhood and adolescent cancers typically exhibit shorter incubation periods and more rapid dissemination. These cancers are generally invasive but tend to be more responsive to chemotherapy^1^. Strategies to enhance early diagnostic capabilities should be implemented to facilitate appropriate treatment options and to increase the probability of survival while optimizing quality of life.

Given that the signs and symptoms of childhood and adolescent cancers frequently mimic those of common benign conditions in this age group, medical professionals must exercise rigorous scrutiny to promptly recognize the presence of neoplasia. In this context, the presence of a pediatrician at all levels of healthcare networks becomes a top priority. In instances of clinical suspicion, patients should be promptly referred to a specialized pediatric oncology center^1,2^.

In this context, numerous studies have evaluated the factors contributing to delayed diagnosis of pediatric cancer, categorizing them based on the following^3^:

- Disease characteristics: onset, tumor site, and aggressiveness of the neoplasm;
- Patient/parental attributes: patient age, ethnicity, parental educational level, parental occupation, and family religious background;
- Accessibility to healthcare.

Regarding patient age, older children, who are less frequently supervised during activities such as dressing or showering, may experience delayed recognition of the signs and symptoms of the disease. Additionally, teenagers may be reticent to discuss health-related concerns with their parents or caregivers, which could impede early diagnosis^3,4^.

## EPIDEMIOLOGY

According to the International Agency for Research on Cancer (IARC), approximately 280,000 children and adolescents between the ages of 0 and 19 years were diagnosed with cancer globally in 2020. Of these, an estimated 110,000 succumbed to the disease. The actual incidence may be significantly higher, given that many countries encounter challenges in accurately diagnosing childhood cancers^5^.

In Brazil, the National Cancer Institute (INCA) projects an estimated 7,930 new cases of childhood and adolescent cancer annually for the 2023–2025 triennium. This corresponds to an estimated risk of 134.81 cases per million children and adolescents. Annually, new cases are estimated to be 4,230 among males and 3,700 among females. These figures translate to an estimated risk of 140.50 new cases per million male children and 128.87 new cases per million female children^6^.

In Brazil, neoplasms in infants constitute 6.3% of all cancer cases among patients in the 0–14 years age group. In this age group, the most diagnosed types of cancer are provided in descending order of frequency: neuroblastoma, leukemia, CNS tumors, retinoblastoma, germ cell tumors, sarcomas, renal tumors, and hepatoblastomas^7,8^.

The biological behavior of cancer in adolescents diverges from that observed in both childhood and adult malignancies. These biological variants encompass divergent genetic risks, histological subtypes, as well as distinct pathways for oncogenic activation and regulation. The cancer in adolescents constitutes 2–6% of the total number of cancer cases. The histological types most encountered in teenagers are Hodgkin lymphoma, osteosarcoma, and testicular cancer^9^.

In terms of anatomical location, specifically in the head and neck region, retinoblastoma, neuroblastoma, and rhabdomyosarcoma are more frequent in infants up to 3 years. In children aged 3–11 years, lymphoma and rhabdomyosarcoma are the most common, while among teenagers and young adults, lymphoma and soft tissue sarcomas prevail^3^.

Globally, more than 100,000 children and teenagers under the age of 20 years succumb to cancer annually. Of these, approximately 75,000 are children aged 0–14 years, while around 27,000 are adolescents aged 15–19 years. The mortality rate is generally lower in high- and middle-income countries, largely attributable to more extensive access to precise diagnostic tests and treatment modalities^2^.

In Brazil, although cancer in children and adolescents is relatively rare, it represents the leading cause of death due to disease, not considering causes due to accidents and violence, among individuals aged 1–19 years^1^.

Trends in mortality rates are influenced by fluctuations in both incidence and survival rates, which, in turn, are shaped by the healthcare system's effectiveness in cancer management, including capabilities for early diagnosis and access to efficacious treatments^1^.

## RISK FACTORS

Most childhood cancer cases are not attributed to hereditary DNA mutations but rather arise from DNA alterations that occur early in the child's life, occasionally even prenatally. These mutations are somatic in nature, localized exclusively to the neoplastic cells, and are not heritable by offspring^1,2,10^.

In childhood and adolescent cancer, there is no scientific evidence to support a significant etiological role for environmental or exogenous factors. However, high dose ionizing radiation and prior chemotherapy are recognized causes. For a substantial number of childhood cancer cases, existing research on etiology is either inconsistent or insufficient to permit a meta-analysis^2,10^.

The minority of childhood cancer cases (5–10%) are caused by inherited predisposition. However, the percentage of inherited contribution varies significantly by cancer type and may be compounded by other genetic factors, as observed in adrenocortical carcinoma, choroid plexus carcinoma, optic nerve glioma, and retinoblastoma^2^.

The chromosomal syndromes most associated with an elevated risk of neoplasm development include Trisomy 21, WAGR syndrome, and syndromes related to chromosomal instability, such as ataxia telangiectasia, Fanconi anemia, Bloom syndrome, Nijmegen syndrome, dyskeratosis congenita, and xeroderma pigmentosum^1,2^.

Advances in genomics underscore the importance of conducting meticulously designed studies to identify the risk factors for cancer in childhood and adolescence, facilitated by the development of innovative analytic techniques. However, such studies necessitate larger sample sizes, which can only be attained through enhanced collaborative efforts^2^.

In this context, in the Brazilian state of Paraná, the incidence of tumors in the adrenal cortex in children is 15–18 times higher compared with that in the United States and Europe. Additionally, in the states of Santa Catarina and São Paulo, this neoplasm has also been observed with greater frequency. The higher incidence is associated with an R337H mutation of the *p53* tumor suppressor gene. It is crucial for doctors in general and pediatricians specifically, to remain vigilant for early signs of puberty, particularly during the first years of life, to diagnose adrenal carcinoma in its initial stages—when surgical intervention alone has a near 100% survival rate^1^.

## DIAGNOSIS

Given the current minimal evidence supporting a significant etiological role for environmental or exogenous factors in childhood cancer, it is imperative to prioritize early diagnosis. However, if medical professionals do not consider the possibility of cancer, this could lead to delayed diagnosis. As a form of secondary prevention, cancer screening in children is either ineffective or applicable only to a limited subset of patients^1,2^.

The exclusion of childhood and adolescent cancer as a diagnostic consideration often leads to delayed diagnosis in various neoplasias, such as bone tumors. In our experience, patients with osteosarcoma typically take an average of 4–5 months to seek medical attention for pain symptoms originating from tumors. In some instances, this period can extend up to 1 year, marked by a diagnostic odyssey involving different medical consultations.

In this context, medical professionals must exercise heightened vigilance, giving special attention to certain signs and symptoms (Table 1) that may be associated with at least 85% of childhood and adolescent cancer cases^1,3^.

**Table 1:** Signs and symptoms of childhood and adolescent cancer.

Signs and symptoms	Possibility
Volume increase in soft tissue (although a history of trauma is frequently observed, no causal relationship has been established)	Sarcoma, leukemia
Increase in testicle volume	Leukemia, germ cell tumor
Persistent morning headaches, possibly associated with neurological alterations, diabetes insipidus, neurofibromatosis, or prior leukemia radiation therapy	Central nervous system tumor Langerhans cell histiocytosis
Abdominal pain, abdominal mass	Solid tumors, differentiation from hepatosplenomegaly
Refractory odontalgia	Lymphoma, rhabdomyosarcoma
Back pain that exacerbates in a supine position, with or without signs of spinal cord compression	Lymphoma, neuroblastoma, primitive neuroectodermal tumor, rhabdomyosarcoma, leukemia
Bone or joint pain, particularly if persistent and causing nocturnal awakening in the child, may be accompanied by edema, a palpable mass, or functional limitation	Leukemia, malignant bone tumor, and neuroblastoma
Ecchymoses, petechiae, and other forms of hemorrhage	Spinal cord involvement by leukemia, lymphoma, and neuroblastoma
Strabismus, nystagmus	Retinoblastoma
Excessive weight gain	Adrenocortical carcinoma
Exophtalmos, palpebral ecchymosis	Neuroblastoma (raccoon eyes), rhabdomyosarcoma, Langerhans cell histiocytosis
Fever of unknown origin persisting over an extended period	Lymphoma, leukemia, neuroblastoma, Ewing sarcoma
Hematuria, sistemic hypertension	Wilms tumor
Hepatosplenomegaly	Leukemia, lymphoma
Heterochromia, anisochromia	Neuroblastoma
Leukocoria or “white pupillary reflex”	Retinoblastoma
Asymmetric lymphadenopathy, similar to “potato bag”	Hodgkin lymphoma
Low cervical lymphadenopathy in teenagers	Thyroid carcinoma
Lymphadenopathy, particularly in the posterior auricular, epitrochlear, and supraclavicular regions	Leukemia and lymphoma
Altered nevi, particularly in areas subject to friction or sun exposure	Melanoma (rare in children)
Chronic otalgia and/or otorrhea, particularly if associated with seborrheic dermatitis	Langerhans cell histiocytosis, rhabdomyosarcoma
Pallor, fatigue	Anemia secondary to spinal cord involvement
Unexplained weight loss	Hodgkin lymphoma, Ewing sarcoma
Pruritus, nocturnal hyperhidrosis	Hodgkin lymphoma
Precocious pseudopuberty	Adrenocortical carcinoma
Vaginal bleeding	Rhabdomyosarcoma
Chronic non-productive cough	Leukemia or lymphoma, with mediastinal mass

Source: Silva et al.^1^.

It is relevant to conduct a detailed clinical history, based on the chief complaint, as well as a thorough physical examination to assist in the identification of the disease in its early stages. Family history and the presence of genetic or constitutional diseases can also aid in diagnostic formulation^1,2^.

Some situations, such as oncological emergencies and urgencies, can manifest as initial symptoms of the disease, develop during treatment, or occur during its progression or recurrence. These conditions include hyperleukocytosis, tumor lysis syndrome, superior vena cava syndrome, superior mediastinal syndrome, intracranial hypertension, spinal cord compression, and febrile neutropenia. These oncological emergencies and urgencies require rapid identification and appropriate treatment to minimize mortality and sequelae in this patient population^1,3^.

In retinoblastoma, the most commonly occurring intraocular tumor in children, diagnosis is confirmed through retinal examination with dilated pupil and specific findings in imaging exams. Direct biopsy of the tumor is contraindicated due to the risk of disease dissemination^1,3^.

In malignant neoplasms requiring biopsy, several general principles should be adhered to for accurate diagnosis: obtaining sufficient tissue without jeopardizing subsequent therapy, favoring excisional biopsy when malignancy involves an organ or lymph node, and ensuring proper preservation of the biopsy material^1,3^.

The use of minimally invasive techniques is increasing, with successful results for both diagnostic tissue acquisition and research investigations. Fine-needle aspiration biopsy (FNAB) is commonly employed in adult cancer diagnosis. However, limitations regarding sample size and the potential need for repeat procedures restrict its utility among children^3^.

Percutaneous image-guided needle biopsy, facilitated by either ultrasound or CT scan, is increasingly being employed for the diagnosis of malignant tumors in pediatric patients, particularly when complete tumor resection is unfeasible. Numerous studies have demonstrated that this approach is accurate, safe, and cost-effective for diagnosing solid tumors in pediatric populations^3^.

Alterations in the hemogram, such as leukocytosis or leukopenia—primarily associated with neutropenia or pancytopenia—may indicate neoplastic infiltration of the bone marrow. These are typically observed in conditions such as leukemia, lymphoma, and neuroblastoma and less commonly in retinoblastoma^1,3^.

Indications for bone marrow aspiration (myelogram) include the following^1,3^:

1. Significant and unexplained reduction of one or more hematologic lineages;
2. Presence of blasts or leukoerythroblastic alterations in peripheral blood;
3. Unexplained association with lymphadenopathy or hepatosplenomegaly;
4. Association to anterior mediastinal mass.

A crucial consideration for all doctors is to avoid corticosteroids prior to establishing a definitive diagnosis, as these drugs may obscure clinical presentation, select for resistant leukemia cells, and adversely affect patient prognosis^1^.

## TREATMENT AND PROGNOSIS

Many pediatric malignancies have high cure rates, and early diagnosis in certain histological subtypes may correlate with improved prognosis, reduced therapy intensity, and fewer disease- or treatment-related complications^3^.

Therapeutic options are diverse, encompassing chemotherapy, surgery, radiation therapy, immunotherapy, targeted therapy, hematopoietic stem-cell transplantation, and organ transplantation. Each modality should be individualized based on the histological type and clinical extent of the disease^1^.

Significant variability exists in survival rates among children and teenagers diagnosed with different neoplasms, depending on factors such as natural history, affected organ, extent of dissemination, and responsiveness to antineoplastic therapies. With advancements in technology, treatment is becoming increasingly individualized through the application of precision medicine and the development of targeted therapy^1^.

In response to the need for cancer management across all age groups, including children, the World Health Organization (WHO) launched the Global Initiative for Childhood Cancer Control in 2018, supported by IARC and other global partners. The initiative aims to achieve a minimum global survival rate of 60% by 2030^1,11^.

Avoidable deaths from childhood and adolescent cancer in low- and middle-income countries can be attributed to factors such as underdiagnosis, delayed or incorrect diagnoses, limited healthcare access, treatment abandonment, higher rates of treatment-related toxicity, and increased recurrence^5,11^.

## CONCLUSION

Although survival rates are contingent on histological diagnosis, 80–85% of all childhood and adolescent cancer types have the potential for cure if detected early and treated at specialized pediatric oncology centers that adhere to cooperative protocols.

If cancer is suspected in a child or teenager, prompt referral to a specialized center is essential for timely diagnosis, clinical staging, and immediate treatment initiation, given that early intervention can mitigate morbidity and disease-related complications.
